# Editorial Note

**Published:** 1950-10

**Authors:** 


### Editorial

Book Reviews have been a regular feature of this *Journal* from the first number. The practice was early established that those who reviewed the books should bestow them in the Society's Library, which thus received year by year a constant supply of the newest books and latest editions. The Society, indeed, owes a very substantial debt, not always recognized, to those members who have so long and so freely given their time and energies for the common good.

But we can scarcely continue to expect this when less than a dozen readers are prepared to express any interest in the Reviews, and the cost of printing them exceeds the published price of the books. After this number therefore " Reviews of Books " will cease.

